# First Evidence of *Aedes albopictus* (Skuse) in Southern Chiapas, Mexico

**DOI:** 10.3201/eid0905.020678

**Published:** 2003-05

**Authors:** Mauricio Casas Martínez, José Luis Torres Estrada

**Affiliations:** * Centro de Investigación de Paludismo/Instituto Nacional de Salud Pública, Chiapas, México

**To the Editor:** The mosquito *Aedes albopictus* (Skuse, 1894) was first identified in the Americas in Texas in 1985 (*1*,*2*). That year, this newly introduced species had dispersed widely in Texas and was implicated in the transmission of dengue virus (*3*). Later, the first states in Mexico that were infested by *Ae. albopictus* were along the northern Mexican border: Coahuila, Nuevo Leon, and Tamaulipas (*4**,**5*; J.P. Martínez-Muñoz, thesis). In 1997, this species was reported farther south in Veracruz (*6*). Although *Ae. albopictus* was expected to spread to southernmost Mexico, this mosquito has never been reported there until now. We have confirmed *Ae. albopictus* in the city limits of Tapachula, southern Chiapas, Mexico.

On September 13, 2002, one of the authors, who resides in Tapachula, was bitten by a mosquito. He collected the specimen, which was later identified as *Ae. albopictus* by the Centro de Investigación de Paludismo (CIP). Nearby larval habitats were then comprehensively searched to collect the immature stages of the species; the sampling area was located at 14° 55' 22.5" north and 92°15' 05.7" west at an altitude of 220 m along the periphery of Tapachula. We found the following containers with larval stages of mosquitos: five water containers, two discarded tires (containing 300–3,000 mL of water), one thermal bottle (250 mL), one plastic bottle (50 mL), and one bucket (2,500 mL). Larvae were placed in plastic bags and transported to CIP laboratories, where they were allowed to emerge to adults during 17 days. The fourth instar larval and pupal exuvias were fixed and identified to species according to Darsie (*7*) and Superintendência de Campanhas de Saúde Pública (*8*). Twenty-five female and male *Ae. albopictus* from these collections are available from CIP laboratory upon request.

Additional field collections are being conducted to establish the distribution range of this species along the Chiapas coastal plain, to determinate the entomologic levels of infestation, and to determine its susceptibility to insecticides. Considering the epidemiologic relevance of this discovery, we have notified the proper health authorities to take necessary control measures to reduce the possibility of increased dengue transmission and to prevent other arboviruses, such as West Nile virus (*9*), from being spread by this new species in southern Mexico.
